# Enzyme-Assisted Extraction Optimization, Characterization and Antioxidant Activity of Polysaccharides from Sea Cucumber *Phyllophorus proteus*

**DOI:** 10.3390/molecules23030590

**Published:** 2018-03-06

**Authors:** Yujing Qin, Qingxia Yuan, Yuexing Zhang, Jialu Li, Xinjiao Zhu, Lingling Zhao, Jing Wen, Jikai Liu, Longyan Zhao, Jinhua Zhao

**Affiliations:** 1 School of Pharmaceutical Sciences, South-Central University for Nationalities, Wuhan 430074, China; 15927271905@163.com (Y.Q.); qingxiayuan@mail.scuec.edu.cn (Q.Y.); zhangyx88@csu.edu.cn (Y.Z.); lijialu@tongji.edu.cn (J.L.); zhu162164131@163.com (X.Z.); 15035297217@163.com (L.Z.); liujikai@mail.scuec.edu.cn (J.L.); 2 College of Life Science and Technology, Lingnan Normal University, Zhanjiang 524048, China; jw82123@126.com; 3 State Key Laboratory of Phytochemistry and Plant Resources in West China, Kunming Institute of Botany, Chinese Academy of Sciences, Kunming 650201, China

**Keywords:** sea cucumber, polysaccharide, enzyme-assisted extraction, characterization, antioxidant activity

## Abstract

Enzyme-assisted extraction optimization, characterization and in vitro antioxidant activity of polysaccharides from sea cucumber *Phyllophorus proteus* (PPP) were investigated in the present study. The optimal extraction conditions with a yield of 6.44 ± 0.06% for PPP were determined as follows: Extraction time of 2.89 h, ratio of extraction solvent to raw material of 16.26 mL/g, extraction pH of 6.83, exraction temperature of 50 °C and papain concentration of 0.15%. Three purified fractions, PPP-1a, PPP-1b and PPP-2 with molecular weights of 369.60, 41.73 and 57.76 kDa, respectively, were obtained from PPP by chromatography of FPA98Cl and Sepharose CL-6B columns. Analysis of monosaccharide compositions showed that PPP-1a consisted of *N*-acetyl-galactosamine (GalNAc), galactose (Gal) and fucose (Fuc), PPP-1b of Fuc as the only monosaccharide and PPP-2 of glucuronic acid, GalNAc and Fuc. Sulfate contents of PPP, PPP-1a, PPP-1b and PPP-2 were determined to be 21.9%, 20.6%, 25.2% and 28.0% (*w*/*w*), respectively. PPP and PPP-1a had higher molecular weight and intrinsic viscosity than those of the PPP-1b and PPP-2. PPP, PPP-1a, PPP-1b and PPP-2 exhibited obvious activities of scavenging 1,1-diphenyl-2-picrylhydrazyl radical, hydroxyl radical, superoxide radical and ABTS radical in different extent, which suggested that the polysaccharides from *Phyllophorus proteus* may be novel agents having potential value for antioxidation.

## 1. Introduction

Sea cucumbers belong to Echinodermata, Holothuroidea, and are important marine invertebrates found in most benthic marine habitats and deep seas across the world [1]. These animals have been a traditional tonic food and folk medicine in many countries for centuries and have gained increasing interest among researchers in recent years for their health benefits and diverse biological activities including anticancer, anti-diabetes, anti-inflammation, anti-obesity, immunomodulatory activity and anti-atherosclerosis [2,3,4,5]. These biofunctions of sea cucumbers can be attributed to their series of bioactive compounds such as triterpenes, lactones, cerebrosides, organic acids and polysaccharides [6,7,8].

Polysaccharides from sea cucumber, as one of its main components, can be divided into two main types, i.e., fucosylated glycosaminoglycan and sulfated fucoidan [9,10]. These biological macromolecules have shown multiple pharmacological activities such as anti-hyperlipidemia, anti-hyperglycemia, antitumor, antivirus, anti-inflammation, anticoagulation and antithrombosis, and may therefore serve as promising agents for some disease treatment and prophylaxis [11,12,13,14]. There are different techniques to extract the polysaccharides such as hot water extraction, microwave-assisted extraction, ultrasound-assisted extraction, and enzyme-assisted extraction [15,16]. Compared with other strategies, enzyme-assisted extraction has the advantage of being easily operated, highly efficient, environmentally friendly, and easily keeping structure of polysaccharides [17]. Enzymes can effectively lyse cells, to release the bioactive components contained inside the cells. Thus, the enzyme-assisted extraction technology has been widely used in natural product extraction. Particularly, in previous studies, researchers found that papain, a cysteine protease, can be applied to release polysaccharides from the proteoglycans in body wall of sea cucumbers [10,13]. However, as for enzyme-assisted extraction of the polysaccharides from sea cucumber, the optimum extraction conditions such as the optimal reaction temperature, extraction time, pH, enzyme concentration and ratio of solvent to raw material are still unclear. Therefore, to extract the polysaccharides efficiently, optimization of the extraction conditions is necessary. The response surface methodology (RSM) is a robust method for optimization of extraction of various active substances and has been used to optimize the extraction conditions of many polysaccharides derived from different species of plant, animal and fungus for its less laborious and time-consuming advantages than other methods [15,16,17]. This method may also be used for optimization of the enzyme-assisted extraction conditions of polysaccharides from sea cucumber.

The polysaccharides from various sea cucumber species have obviously distinctive structures such as sulfate substitution patterns and monosaccharide proportions, and different physicochemical characteristics [18,19]. Some uncommon sea cucumber species remain to be studied, one of which is *Phyllophorus proteus*, a seafood consumed in China. The anticoagulant and antithrombotic activities of sea cucumber polysaccharides have been extensively studied, whereas, their antioxidant activities activity is rarely investigated [14]. It was reported that the extracts from *Parastichopus tremulus* and *Holothuria forskali* and the crude polysaccharides from *Apostichopus japonicas* had anti-oxidant activity [20,21,22]. While little information on the polysaccharides from sea cucumber *Phyllophorus proteus* (PPP) is available. Therefore, to study the structural characteristic of PPP and their antioxidant activities may be worthy to expand the knowledge and possible application of sea cucumber polysaccharides.

In the present study, we report here the results of enzyme-assisted extraction optimization, the physicochemical characteristics and in vitro antioxidant activity of PPP. RSM was employed to optimize the extraction conditions, and PPP were prepared by the optimal conditions selected. The purified fractions isolated from PPP were then characterized via chemical analysis, chromatography and spectroscopy methods. Finally, the antioxidant activities of PPP and its purified fractions were investigated. 

## 2. Materials and Methods

### 2.1. Materials and Chemicals

The dry sea cucumber *Phyllophorus proteus* was collected from Zhanjiang City (Guangdong, China), and identified by one of coauthors, professor Wen using the mitochondrial *cytochrome oxidase I* (*COI*) gene as molecular marker [23]. The partial *COI* gene sequence of the collected sea cucumber determined was: ATAATGATTAAGAGGATTTGGAAACTGACTTATCCCATTAATGATAGGAGCCCCAGATATGGCTTTTCCCCGAATGAAAAAAATGAGATTCTGACTAGTTCCCCCATCCTTTATCTTACTATTAGCCTCAGCTGGTGTAGAAAGAGGAGTAGGAACAGGATGAACAATATATCCACCCTTATCAAGAAAAATTGCTCATGCAGGAGGATCCGTCGATCTTGCCATTTTCTCTCTCCACCTAGCAGGAGCCTCATCAATCCTAGCCTCAATAAACTTCATCACCACCATAATAAAAATGCGATCCCCAGGAATTAGATTTGATCGCCTACCACTATTCATCTGATCAGTCTTCATAACAGCTTTCCTCCTCCTCCTAAGACTACCAGTCCTAGCAGGAGCCATAACTATGCTTCTAACCGACCGAAAAATCAAAACAACATTCTTTGACCCCTCAGGAGGAGGAGATCCTATACTATTTCAACACCTATTCTGATTCTTTGGACACCCAGAAGTCTACATTCTTATCCTACCAGGATTCGGAATGATATCCCACATAGTTGCCCACTACAGCGGAAAGCAAGAACCCTTCGGATATCTAGGAATGGTCTACGCAATGGTAGCCATTGGCATTTTAGGCTTCCTTGTATGAGCACACCACATGTTTACTGTTGGAATGGATGTAGCAACCACGAGCA. The homogeny between this sequence and the known sequence of *Phyllophorus proteus* in the international Bio-barcode System (BOLD SYSTEMS) was up to 99%.

Amberlite FPA98Cl was obtained from H&E Co., Ltd. (Beijing, China). Sepharose CL-6B was purchased from GE Healthcare Life Sciences (Uppsala, Sweden). Papain (800 U/mg) was purchased from Shanghai Yuanye Bio-Technology Co., Ltd. (Shanghai, China). Standard D-series Dextrans (D-2, 3, 4, 5, 6, 7 and 8) were obtained from National Institutes for Food Drug Control (Beijing, China). l-Rhamnose (Rha), d-galactose (Gal), d-galacturonic acid (GalA), d-glucose (Glc), d-glucuronic acid (GlcA), 3-methyl-1-phenyl-2-pyrazolin-5-one (PMP), 1,1-diphenyl-2-picrylhydrazyl (DPPH), ascorbic acid (Vc), phenazine methosulphate (PMS), nitroblue tetrazolium (NBT), reduced nicotinamide adenine dinucleotide (NADH) and [2,2′-azinobis-(3-ethyl-benzothiazolin-6-sulfonic acid)] diammonium salt (ABTS) were purchased from Sigma Chemical Co. (St. Louis, MO, USA). d-Mannose (Man), l-arabinose (Ara), d-ribose (Rib), d-xylose (Xyl), *N*-acetyl-galactosamine (GalNAc) and l-fucose (Fuc) were purchased from Aladdin Chemical Reagent Co., Ltd. (Shanghai, China). All other chemicals used were of analytical grade.

### 2.2. Extraction of Polysaccharides

The dry sea cucumbers were further dried by hot air at 60 °C for 24 h, ground into fine powder and sieved through a 60-mesh sieve to obtain sea cucumber powder. Five grams of the dried powder was used in each extraction process, and extracted under experimental condition. After extraction, the suspension was adjusted to pH 2.8 by 1 M HCl, kept at 4 °C for 4 h and centrifuged at 7441× *g* for 20 min to remove acidic protein. The supernatant was neutralized with 1 M NaOH, mixed with a triple volume of absolute ethanol and kept at 4 °C for 4 h. The precipitates were collected by centrifugation at 4816× *g* for 15 min and freeze-dried to obtain PPP. The extraction yield was calculated by the following formula: 

Extraction yield (%) = W_1_/W_0_ × 100, where W_1_ and W_0_ are the weights of PPP and the dried sea cucumber sample respectively.

### 2.3. Experimental Design of RSM

To optimize the production of PPP, extraction conditions that may affect its extraction yield such as temperature, extraction time, pH, enzyme concentration of papain and ratio of extraction solvent to raw material were investigated using single-factor test method. At the beginning of the single-factor tests, temperature, extraction time, pH, enzyme concentration and ratio of extraction solvent to raw material were set at 50 °C, 6 h, 6.5, 0.2%, and 15 mL/g, respectively, according to our previous study [10]. The experimental conditions were the same in each test except the investigated factor, which was changed. On the basis of the single factor experiments, the factors having significant impact on PPP yield were screened out for the response surface experiments. The three factors chosen in our present study were the ratio of extraction solvent to raw material (mL/g), extraction time (h) and extraction pH, which were designated as *X*_1_, *X*_2_ and *X*_3_, and prescribed into three levels, coded −1, 0, and +1 for low, middle and high value, respectively (Table 1). The test variables were coded according to the following Equation (1):

(1) $$X_{i}=\frac{\chi_{i}-\chi_{0}}{\Delta \chi_{i}}$$

where *X*_i_ and *χ_i_* are the coded and actual value of the independent variable, respectively, *χ*_0_ is the actual value of the independent variable at the central point, and Δ*χ_i_* is the step change of the variable.

The extraction yield of PPP was defined as the dependent variable. The Box–Behnken design consisted of 17 experimental points, each of which was carried out in triplicate in a random order.

To predict the optimal point, a quadratic polynomial model was fitted to correlate relationship between the independent variables and response (PPP yield), as shown below Equation (2):

(2) $$Y=\beta_{0}+\sum_{i=1}^{3}\beta_{i}X_{i}+\sum_{i=1}^{3}\beta_{i}{}_{i}X_{i}^{2}+\sum_{i=1}^{2}\sum_{j=i+1}^{3}\beta_{i}{}_{j}X_{i}X_{j}$$

where *Y* is the response variable (extraction yield of PPP); *β_0_, β_i_, β_ii_* and *β_ij_* are the regression coefficients of variables for intercept, linear, quadratic and interaction terms, respectively; *X*_i_ and *X*_j_ are the independent variables (*i* ≠ *j*).

### 2.4. Isolation of Homogeneous Polysaccharides from PPP

PPP was dissolved in deionized water and loaded onto an FPA98Cl column (4.0 × 50 cm). Then, the column was stepwise eluted with 0, 0.5, 1.0, 1.5 and 2.0 M sodium chloride (NaCl) solution at a flow rate of 2 (BV) /h. Then obtained fraction PPP-1 or PPP-2 was loaded onto a Sepharose CL-6B column (2.0 × 150 cm) and eluted with 0.2 M NaCl aqueous solution at a flow rate of 15 mL/h. The eluents were collected (10 mL/fraction) with an automatic fraction collector. Three completely separated fractions, PPP-1a, PPP-1b and PPP-2, were collected by checking the absorbance at 490 nm using phenol-sulphuric acid method [24], dialyzed against distilled water for 3 days with a dialysis membrane (molecular weight cut-off of 3500 Da) and lyophilized.

### 2.5. Characterization of PPP, PPP-1a, PPP-1b and PPP-2

#### 2.5.1. Determination of Homogeneity and Molecular Weight

The homogeneity and molecular weight of PPP, PPP-1a, PPP-1b and PPP-2 were examined by high-performance gel permeation chromatography (HPGPC) using an Agilent 1260 series apparatus (Agilent Technologies, Santa Clara, CA, USA) equipped with a RID detector and a Shodex OH-pak SB-804 HQ or Shodex OH-pak SB-806 HQ column (8 mm × 300 mm). The column was eluted with 0.1 M NaCl solution at a flow rate of 0.5 mL/min at 35 °C. The HPLC system was calibrated by D-series dextran standards (D-2, D-3, D-4, D-5, D-6, D-7 and D-8) [9].

#### 2.5.2. Analysis of Monosaccharide Composition, Protein and Sulfate Content

The monosaccharide compositions of PPP, PPP-1a, PPP-1b and PPP-2 were analyzed according to the reported method [15] with some modifications. Briefly, the monosaccharide standards and polysaccharide solution (100 μL, 5 mg/mL) were hydrolyzed with 100 μL of 4 M trifluoroacetic acid (TFA) at 110 °C for 4 h under the nitrogen environment. After removing the excess TFA, the hydrolysate was labeled with PMP and analyzed by an Agilent 1260 HPLC system equipped with a DAD detector and an Eclipse Plus C18 column (4.6 × 250 mm, 5 µm, Agilent). The injection volume was 20 μL, the mobile phase was a mixture of phosphate buffered saline (PBS, 0.1 M, pH 6.7) and acetonitrile in a ratio of 83: 17 (*v*/*v*), column temperature was 30 °C, flow rate was 1.0 mL/min, and detector wavelength was 245 nm.

The protein content was measured by the Bradford analysis, as previous study [15]. The content of sulfate radical was determined according to the BaCl_2_/gelatin method as described previously [25]. The SO_3_^−^ to COO^−^ molar ratios of the polysaccharides were determined by a conductimetric titration method [26].

#### 2.5.3. NMR Analysis

All purified fractions were dissolved in deuterium oxide (D_2_O, 99.9% D) and lyophilized three times to replace exchangeable protons with D_2_O. The lyophilized samples were then dissolved in D_2_O at a concentration of 40 mg/mL. NMR analyses were performed at 298 K with a 600 MHz Bruker Advance spectrometer (Zurich, Switzerland) in the FT mode as described previously [27].

#### 2.5.4. Specific Rotation, Intrinsic Viscosity and FT-IR Spectrometric Analysis

The specific rotation of the polysaccharides was detected by a polarimeter at the concentration of 2 mg/mL at 20 °C. The intrinsic viscosity [η] was determined in a 0.1 M NaCl solution at 25 °C using an Ubbelohde-type capillary viscometer according to the method in Pharmacopoeia of the People’s Republic of China [28]. The purified fractions PPP-1a, PPP-1b and PPP-2 were mixed with spectroscopic-grade potassium bromide powder, ground and pressed into pellets for FT-IR measurement. FT-IR spectra were recorded on a Bruker Tensor 27 infrared spectrometer (Ettlingen, Germany) at the frequency range of 4000–400 cm^−1^.

### 2.6. Assay of In Vitro Antioxidant Activities 

#### 2.6.1. Assay of DPPH Radical Scavenging Activity

The DPPH radical scavenging activity of polysaccharide samples were carried out according to the method of previous report with slight modifications [29,30]. Briefly, PPP, PPP-1a, PPP-1b or PPP-2 solution was dissolved in deionized water at different concentrations (0.25, 0.5, 1.0, 2.0, and 4.0 mg/mL) and added into a 96-well plate (50 μL/well). The 25 μL of DPPH-ethanol solution (0.4 mM) and 100 μL of deionized water were then pipetted into each well. After reaction for 30 min at room temperature in the dark, the absorbance at 517 nm was detected by a microplate reader (BioTek Instruments Inc., Winooski, VT, USA). Vc was included as positive control. DPPH radical scavenging activity was calculated as Equation (3):

DPPH radical scavenging activity (%) = [1 − (A_1_ − A_2_)/A_0_] × 100 (3)

where A_1_ is the absorbance of the samples, A_2_ is the absorbance of the samples under identical conditions as A_1_ except without DPPH in the reaction system, and A_0_ is the absorbance of the blank control (ultrapure water instead of sample).

#### 2.6.2. Assay of Hydroxyl Radical Scavenging Activity

The reported method [31] with slight modifications was applied to detect the hydroxyl radical scavenging activities of PPP, PPP-1a, PPP-1b and PPP-2, compared with Vc. The reaction mixture was prepared by mixing several different reagents in sequence: 50 μL of ferrosin (0.75 mM), 75 μL of phosphate buffer (0.15 M, pH 7.4), 50 μL of FeSO_4_ (0.75 mM), 50 μL of sample solution and 50 μL of H_2_O_2_ (0.01%, *w*/*v*). After 30 min of reaction at 37 °C, the absorbance of the mixture was measured at 536 nm. The scavenging activity was calculated as Equation (4):

Hydroxyl radical scavenging activity (%) = [(A_2_ − A_0_)/(A_1_ − A_0_)] × 100 (4)

where A_2_ is the absorbance of the sample, A_1_ is the absorbance of reaction mixture using ultrapure water instead of H_2_O_2_ and sample, and A_0_ is the absorbance of the blank control (ultrapure water instead of sample).

#### 2.6.3. Assay of Superoxide Radical Scavenging Activity

The scavenging effects of polysaccharides on superoxide radical were determined by a method named PMS/NADH-NBT system as described previously [15,32] with some modifications. The mixtures contained 50 μL of samples (0.25–4.0 mg/mL), 50 μL of NBT solution (156 μM), 50 μL of NADH solution (156 μM) and 50 μL of PMS solution (60 μM), were kept at 25 °C for 5 min. The absorbance was measured at 560 nm with ultrapure water as a blank control. Vc was used as positive control. The scavenging ability was calculated using the following Equation (5):

Scavenging rate (%) = [1 − (A_1_ − A_2_)/A_0_] × 100 (5)

where A_1_ is the absorbance of various samples, A_2_ is the background absorbance of the sample (0.1 M phosphate buffer instead of NBT solution), and A_0_ is the absorbance of the blank control (ultrapure water instead of sample).

#### 2.6.4. Assay of ABTS Radical Scavenging Activity

The ABTS radical scavenging activities of PPP, PPP-1a, PPP-1b and PPP-2 were measured by ABTS radical cation decolorization assay according to the literature procedure [33,34]. Briefly, ABTS solution (7 mM) was oxidized with potassium persulphate (4.95 mM) in the dark at room temperature for 12 h and then diluted with PBS (0.2 M, pH 7.4) to produce an absorbance of 0.70 ± 0.01 at 734 nm. 200 µL of the resulting ABTS^+^ solution was then mixed with 20 µL of sample solution and kept at room temperature for 6 min, and the absorbance at 734 nm was determined. The ABTS radical scavenging activity was calculated as following Equation (6):

ABTS radical scavenging activity (%) = [1 − (A_1_ − A_2_)/A_0_] × 100 (6)

where A_1_ is the absorbance of various samples, A_2_ is the background absorbance of samples (PBS instead of ABTS^+^ solution), and A_0_ is the absorbance of the blank control (ultrapure water instead of sample).

### 2.7. Statistical Analysis

The Design-Expert software version 8.0.6 (Stat-Ease, Inc., Minneapolis, MN, USA) was used for the experimental design and data analysis of RSM. The results of the antioxidant assay were reported as mean ± SD of three replicates. Data were statistically analyzed by One-Way analysis of variance (ANOVA) procedure with SPSS software version 19.0 (IBM SPSS, Inc., Chicago, IL, USA), followed by the Duncan test. *P*-value of less than 0.05 was regarded as significance.

## 3. Results and Discussion

### 3.1. Optimization of Extraction Parameters

#### 3.1.1. Model Building and Statistical Analysis

**Figure 1:**
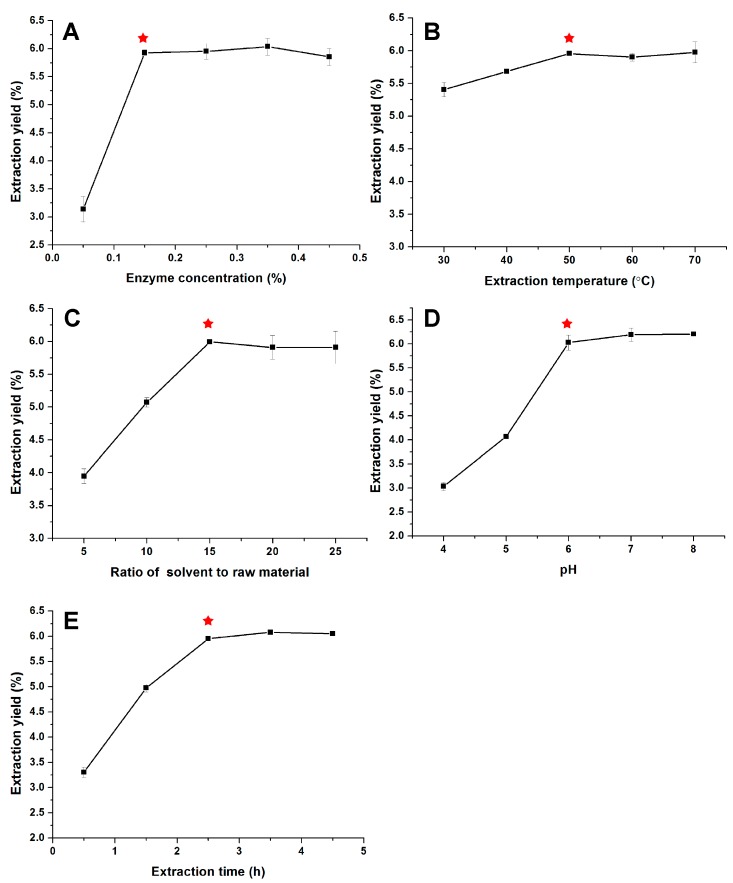
Effects of different extraction parameters such as enzyme concentration (**A**), temperature (**B**), ratio of solvent to raw material (**C**), pH (**D**), and time (**E**) on the yield of polysaccharides from sea cucumber *Phyllophorus proteus*. ★ indicates the optimal reaction condition for each single-factor test.

In our present study, five parameters including enzyme concentration, extraction temperature, extraction time, ratio of solvent to raw material and pH were picked out for investigating their influences on the PPP extraction yields. According to results of the single factor experiments in Figure 1, PPP extraction yield significantly increased (*p* < 0.05) from 3.14% to 5.93% as the enzyme concentration increased from 0.05% to 0.15% (Figure 1A). When the enzyme concentration continued to increase, the extraction yields had no obvious changes (*p* > 0.05). Therefore, 0.15% was chosen as the extracting enzyme concentration through all the extraction optimization experiments. The temperature (30, 40 and 50 °C) (Figure 1B), ratio of solvent to raw material (5:1, 10:1, 15:1) (Figure 1C), pH (4, 5 and 6) (Figure 1D) and extraction time (0.5, 1.5 and 2.5 h) (Figure 1E) affected the PPP extraction yields significantly (*p* < 0.05). Further increasing the level of these factors, the PPP yields decrease or increase sightly (*p* > 0.05). When temperature increased from 30 to 70 °C, the PPP yields increased from 5.41% to 5.98%. Taking into consideration that the effect of temperature was greatly inferior to that of other factors, extraction temperature was not considered in further RSM experiments. Therefore, the three factors, ratio of solvent to raw material, pH, and time, were screened out for the response surface experiments.

The results of the 17 runs including the design and experimental values in BBD design were listed in Table 2. The PPP yield and the test variables were related by the following second-order polynomial Equation (7):

*Y* = −59.32119 + 1.31749*X*_1_ + 5.04959*X*_2_ + 14.85909*X*_3_ − 0.037159*X*_1_*X*_2_ − 0.019837*X*_1_*X*_3_ − 0.076665*X*_2_*X*_3_ − 0.033252*X*_1_^2^ − 0.68269*X*_2_^2^ − 1.10467*X*_3_^2^ (7)

The analysis results of ANOVA, adequacy and fitness of the response surface quadratic model were shown in Table 3. The *F*-value (49.15) and *P*-value (<0.001) turned out that the model was highly significant. The lack of fit values of *F* and *P* were 0.013 and 0.9976, respectively, indicating that the model fitted well. The determination coefficient (*R*^2^) and adjusted determination coefficient (*adj*-*R*^2^) were further applied to check the goodness-of-fit of the model. The value of *R*^2^ was 0.9844, which meant that there was satisfactory correlation between actual and predicted values. The value of *adj*-*R*^2^ was 0.9644, which indicated that most variations (>96%) of the extraction could be predicted by the model. 

In general, a small *p* value less than 0.05 indicated that the corresponding coefficient was significant. Accordingly, the independent variables (*X*_1_, *X*_2_ and *X*_3_) and all three quadratic terms (*X*_1_^2^, *X*_2_^2^ and *X*_3_^2^) can significantly (*p* < 0.001) affect the extraction yield of PPP.

#### 3.1.2. Optimization of Extraction Conditions of PPP and Validation of the Model

**Figure 2:**
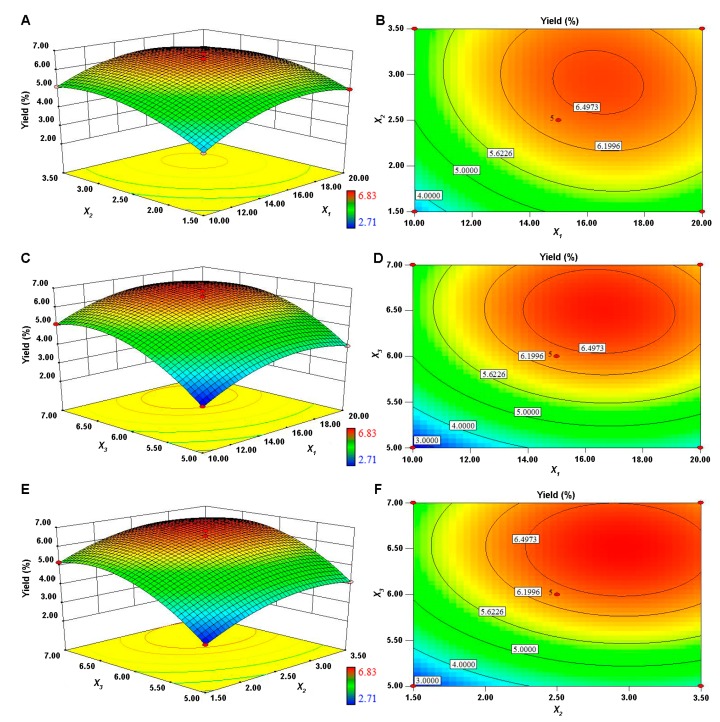
Response surface plots (**A**, **C** and **E**) and contour plots (**B**, **D** and **F**) showing the effects of variables (*X*_1_, ratio of extraction solvent to raw material; *X*_2_, extraction time; *X*_3_, extraction pH) and their mutual effects on the extraction yield of PPP.

The 3D response surface and 2D contour plots of the BBD were obtained using Design Expert software (Version 8.0.6). The responses of PPP yields to changes in ratio of extraction solvent to raw material (mL/g), extraction time (h) and extraction pH were presented in Figure 2. In the 3D response surface and 2D contour plots, the extraction yield of PPP was obtained along with two continuous variables, while the third variable was fixed at level 0. It was obvious that when increased the three variables the PPP yield increased at first, and then gradually decreased. Additionally, the extraction pH had more significant impact on PPP yield than the ratio of extraction solvent to raw material and extraction time. These results were consistent with the ANOVA analysis.

The shapes of the contour plots can reflect the interaction effects between the variables. Elliptical contour plot indicated that the interactions of two independent variables were significant, while the circular contour plots were considered negligible [35,36,37]. According to Figure 2 and Table 3, the interactions between the variables were not significant (*p* > 0.05).

Based on the single factor experiments and the response surface analysis, the optimal extraction conditions of PPP were concluded as follows: papain concentration of 0.15%, extraction temperature of 50 °C, ratio of extraction solvent to raw material 16.26 mL/g, extraction time 2.89 h and extraction pH 6.83. Under the optimal levels of the extraction variables, the maximum response was 6.83%. A mean value of 6.44 ± 0.06% (*n* = 3) was obtained from real experiments, which was well consistent with the predicted value, and demonstrated the validation of the RSM model for the extraction process. Notably, the yield of PPP was higher than that of some crude polysaccharides (1.7%~4.9%) from other sea cucumbers [38,39]. The optimal extraction conditions of PPP established in this study also require less extraction solvent and extraction time compared with those in some researches [10,39]. As stated previously, sea cucumber body wall mainly contains two types of sulfated polysaccharides, i.e., fucosylated glycosaminoglycan and sulfated fucoidan. Therefore, the optimized extraction method may be used for sufficient extraction of polysaccharides from the dried fine powder of other sea cucumber species.

### 3.2. Isolation and Characterization of Polysaccharides from PPP

**Figure 3:**
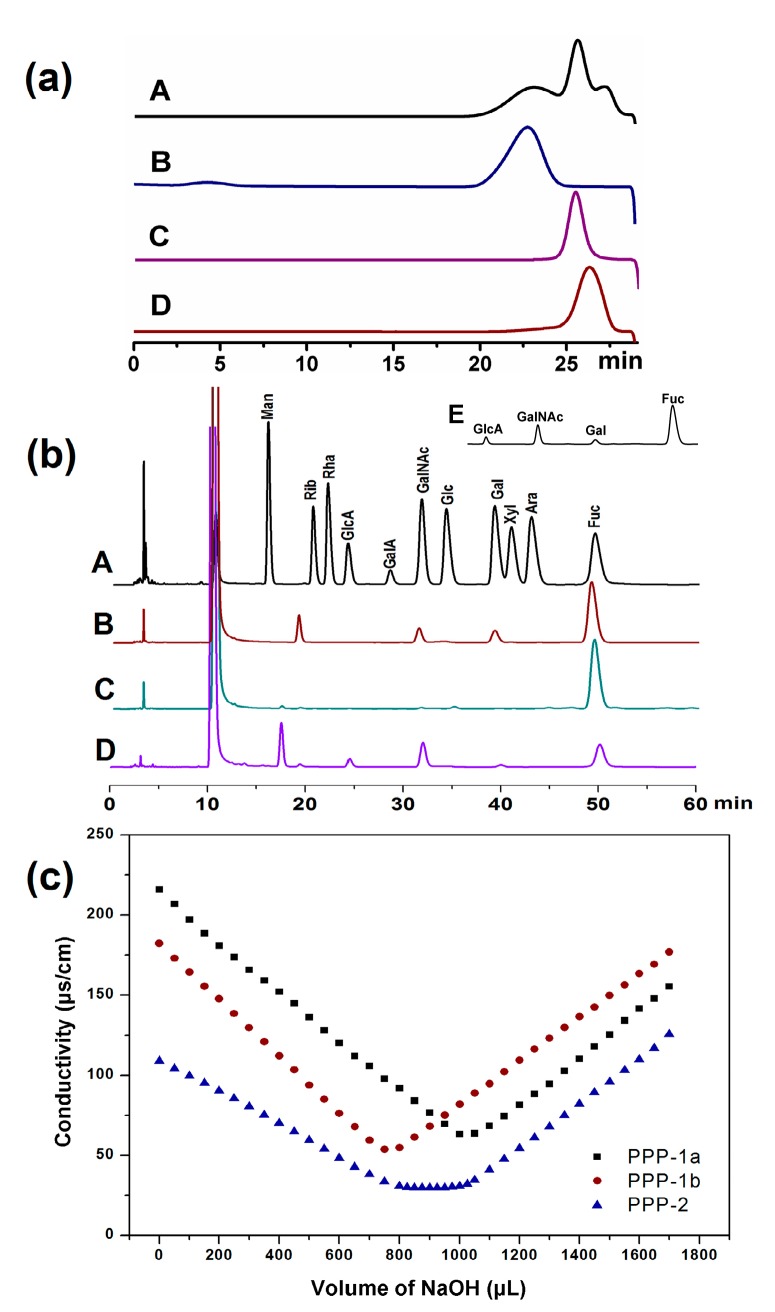
HPLC profiles of PPP (A), PPP-1a (B), PPP-2 (C) and PPP-1b (D) (**a**), chromatograms of PMP derivatives of mixed monosaccharide standards (A), PPP-1a (B), PPP-1b (C), PPP-2 (D) and PPP (E) (**b**), and conductimetric titration curves of PPP-1a, PPP-1b and PPP-2 (**c**).

PPP was prepared using the optimal extraction conditions. Three signal peaks of the extracted polysaccharides were observed in HPGPC spectrum by a Shodex OH-pak SB-806 HQ column (Figure 3). Then, the fractions were separated by an FPA98Cl column, which afforded two independent elution peaks designated as PPP-1 and PPP-2. The PPP-1 fraction was further separated into two components PPP-1a and PPP-1b by a Sepharose CL-6B column (Figure 3). As shown in Table 4, the residual proteins or polypeptides in PPP can be removed effectively by these columns.

#### 3.2.1. Homogeneity and Molecular Weights of PPP, PPP-1a, PPP-1b and PPP-2

The homogeneity and molecular weights of PPP, PPP-1a, PPP-1b and PPP-2 were analyzed by HPLC. As shown in Figure 3a, PPP contained three polysaccharides with different molecular weight ranging from 40 to 400 kDa. A single and symmetrical signal peak was observed for each fraction, indicating that they were homogeneous (Figure 3a). The average molecular weights of PPP-1a, PPP-1b and PPP-2 were estimated to be 369.6, 41.73 and 57.76 kDa (Table 4), respectively, according to the calibration curve.

#### 3.2.2. Monosaccharide Composition of PPP, PPP-1a, PPP-1b and PPP-2

The results of monosaccharide compositions of PPP-1a, PPP-1b and PPP-2 determined by HPLC were showed in Figure 3b and Table 4. It showed that PPP was composed of GlcA, GalNAc, Gal and Fuc with the molar ratio of 1.00:2.34:0.29:9.41, and PPP-1a was composed of GalNAc, Gal and Fuc with the molar ratio of 1.00:1.24:8.04. PPP-1b contained Fuc as the only monosaccharide. PPP-2 was composed of GlcA, GalNAc and Fuc with the molar ratio of 1.00:1.17:1.77. The sulfate contents of PPP, PPP-1a, PPP-1b and PPP-2 were 21.9%, 20.6%, 25.2% and 28.0%, respectively. PPP-1a and PPP-1b only contained sulfate group, while PPP-2 contained both sulfate and carboxyl groups as shown by the conductometric titration (Figure 3c and Table 4). These results indicated that PPP, PPP-1a, PPP-1b and PPP-2 were acidic polysaccharides.

Although various sulfated polysaccharides were obtained from some sea cucumber species [22,40], no polysaccharides with similar monosaccharide composition to PPP-1a were reported as far as we know. Moreover, the molecular weight of PPP-1b was significantly lower than those of the fucoidans isolated from other sea cucumber species [13,19,41,42,43]. Therefore, the sulfated polysaccharides isolated from *Phyllophorus proteus* may be novel polysaccharides with special structures.

#### 3.2.3. Specific Rotation, Intrinsic Viscosity and FT-IR Spectrometric Analysis

The specific rotations of PPP, PPP-1a, PPP-1b and PPP-2 were determined to be −83.22°, −77°, −138.5° and −42.5°, respectively (Table 4). The data showed that the different types of polysaccharides are all levorotatory, although their specific rotations are different. The strongly negative specific rotations of these polysaccharides may be due to the residues of L-fucopyranose. 

Intrinsic viscosity was measured by capillary viscometers of the Ubbelohde type. As shown in Table 4, the intrinsic viscosities [η] of PPP (106.13) and PPP-1a (172.44) were higher than those of the PPP-1b (62.84) and PPP-2 (39.61), which may due to their relative high molecular weights [9].

**Figure 4:**
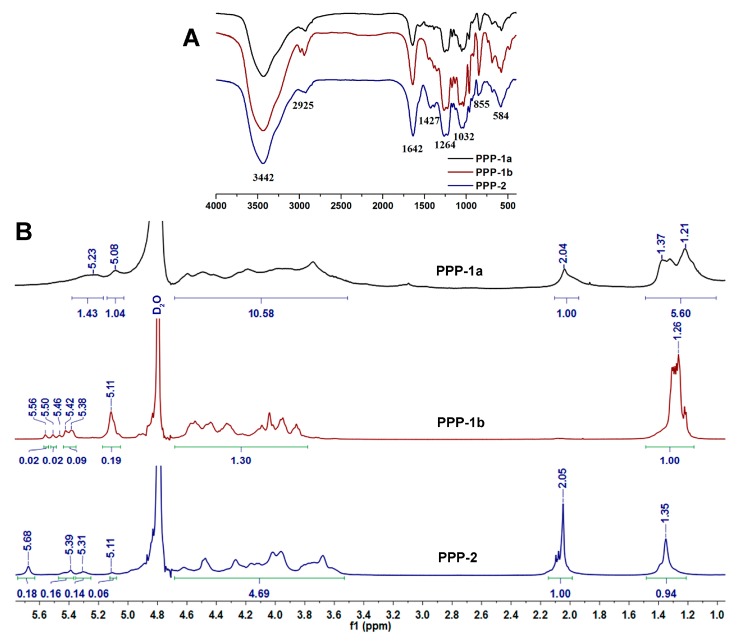
FI-IR (**A**) and ^1^H-NMR (**B**) spectra of PPP-1a, PPP-1b and PPP-2.

The FT-IR spectra of PPP-1a, PPP-1b and PPP-2 were shown in Figure 4A. Broad and strong absorption bands at around 3442 cm^−1^ assigned for the C−H stretching vibrations, 1032 cm^−1^ for the C−O stretching vibrations and 2925 cm^−1^ for the C−H stretching vibrations were observed in the FT-IR spectra of PPP-1a, PPP-1b and PPP-2, respectively. The signal peaks at 1264, 855 and 584 cm^−1^ were assigned to the S=O stretching vibration in sulfate group, C−O−S bending vibration in sulfate group and stretching vibration of S−O, respectively [44]. The absorption peaks at 1642 cm^−1^ and 1427 cm^−1^ for PPP-2 were derived from the stretching vibrations of C=O and COO− in the glucuronic acid, respectively [12,45]. Since PPP-1a and PPP-1b did not contain glucuronic acid, the absorption peak at around 1642 cm^−1^ may be derived from the stretching vibrations of associated water [46,47,48]. These signals observed in FT-IR spectra of PPP-1a, PPP-1b and PPP-2 were in agreement with the results of their chemical composition analysis.

#### 3.2.4. NMR Analysis of PPP-1a, PPP-1b and PPP-2

^1^H-NMR spectra of PPP-1a, PPP-1b and PPP-2 were showed in Figure 4B. The spectra of the polysaccharides (PPP-1a, PPP-1b and PPP-2) from the sea cucumber *Phyllophorus proteus* were quite different from each other and all showed a high purity. The signals of PPP-1a, PPP-1b and PPP-2 at about 1.2–1.4 ppm and 2.0–2.1 ppm corresponded to the methyl protons of Fuc and GalNAc residues, respectively [27]. The signals at around 3.4–4.6 ppm in spectra of the three polysaccharides can be assigned to the cross ring protons [44,49]. And the chemical shifts of the envelope of anomeric signals of the polysaccharides at 5.0–5.7 ppm were consistent with the presence of α-l-fucopyranosyl units [27,50,51]. Specifically, anomeric signals at 5.08 and 5.23 ppm in the spectrum of PPP-1a may be assigned to unsulfated and 2-mono-*O*-sulfo-l-fucopyranosyl residues, respectively, signals at 5.11, 5.38 and 5.56 ppm in the spectrum of PPP-1b may be assigned to unsulfated, 2-mono-*O*-sulfo- and 2,4-di-*O*-sulfo-l-fucopyranosyl residues, respectively, and signals at 5.11, 5.31, 5.39 and 5.68 ppm in the spectrum of PPP-2 may be assigned to unsulfated, 4-mono-*O*-sulfo-, 3-mono-*O*-sulfo- and 2,4-di-*O*-sulfo-l-fucopyranosyl residues, respectively [27,41,52]. No signals were observed at about 2.0–2.1 ppm in the spectrum of PPP-1b, indicating that PPP-1b contained no GalNAc residue. These NMR results were consistent with those of their monosaccharide composition analysis. The NMR analysis of PPP-1a, PPP-1b and PPP-2 showed overlapping spectra with broad signals, which hamper precise resolution, as expected for polysaccharides of high molecular mass. In our previous studies, the precise structures of a fucosylated glycosaminoglycan and a sulfated fucan from *Stichopus variegatus* and *Ludwigothurea grisea*, respectively, have been elucidated by analyzing the structures of their depolymerized products, which contain the repeating trisaccharide unit -{(l-Fuc-α1,3-)d-GlcA-β1,3-d-GalNAc-β1,4-}- and the regular (1→2) and (1→3)-linked tetrasaccharide repeating unit, respectively [10,12]. In the present study, PPP-1a, PPP-1b and PPP-2 had obviously different ^1^H-NMR spectra from those of the previously reported polysaccharides from other species of sea cucumbers [10,12,27,41,44]. The structures of these polysaccharides may be also elucidated by using the depolymerization methods [10,12]. The detailed structures of these polysaccharides will be studied in the near future.

### 3.3. In Vitro Antioxidant Activity of PPP

#### 3.3.1. DPPH Radical Scavenging Activity

**Figure 5:**
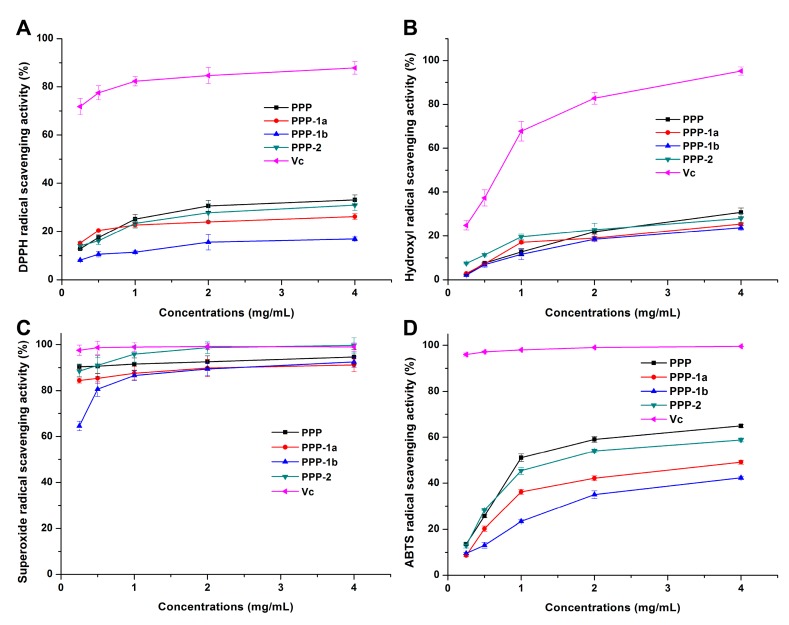
Scavenging effects of PPP, PPP-1a, PPP-1b and PPP-2 on DPPH radical (**A**), hydroxyl radical (**B**), superoxide radical (**C**) and ABTS radical (**D**), respectively.

The assay of DPPH free radical scavenging has been widely used to evaluate the antioxidant activity of natural compounds for its simple, sensitivity, comparable and reproducible operation [53,54]. Antioxidant such as Vc can scavenge DPPH radical by donating a hydrogen to form the stable DPPH–H molecules. It has been reported that the antioxidant activity of polysaccharides was related to electron-withdrawing carboxyl or acetyl groups, which could activate the hydrogen atom of the sugar residues through field and inductive effects [55]. As shown in Figure 5A, PPP, PPP-1a, PPP-1b and PPP-2 all exhibited does-dependently DPPH scavenging activity. At a concentration of 4.0 mg/mL, the DPPH free radical scavenging rates were 33.07%, 26.22%, 16.89%, 30.90% and 87.83% for PPP, PPP-1a, PPP-1b, PPP-2 and Vc, respectively. In the test ranges, DPPH scavenging activities of PPP, PPP-1a and PPP-2 were significantly higher (*p* < 0.05) than that of PPP-1b, which may be due to the carboxyl or acetyl group contents. Proteins or polypeptides in PPP may also increase its scavenging effect on DPPH for these molecules also showed good antioxidant activities [56].

#### 3.3.2. Hydroxyl Radical Scavenging Activity

The effects of PPP, PPP-1a, PPP-1b or PPP-2 on the hydroxyl radical scavenging were detected and compared with the positive control Vc. The result showed that they displayed the activity at a concentration-dependent manner (Figure 5B). At a concentration of 4.0 mg/mL, the hydroxyl radical scavenging rates of PPP, PPP-1a, PPP-1b, PPP-2 and Vc were 30.76%, 25.41%, 23.72%, 27.99% and 95.14%, respectively, and PPP and PPP-2 had slightly higher activities than those of PPP-1a and PPP-1b (Figure 5B). Hydroxyl radicals are speculated to be generated from the Fe^2+^/H_2_O_2_ Fenton reaction system [57]. Previous studies have suggested that hydroxyl radical scavenging activity of polysaccharides may be due to inhibition of hydroxyl radical generation by chelating Fe^2+^ ions [58]. In this study, carboxyl and sulfate groups in PPP, PPP-1a, PPP-1b and PPP-2 are highly nucleophilic and may chelate with Fe^2+^ ions. Consequently, the slightly higher hydroxyl radical scavenging activities of PPP and PPP-2 may be due to their higher content of uronic acid.

#### 3.3.3. Superoxide Radical Scavenging Activity

Superoxide anion can induce lipid peroxidation for it is one of the precursors of the hydroxyl radical and singlet oxygen, which cause oxidative damage to proteins, DNA and enzymes in vivo. Superoxide anion can also magnify the cellular damage and further exacerbate the progression of many diseases including atherosclerosis and arthritis by producing other types of free radicals and oxidizing agents [55]. The superoxide radical scavenging activities of polysaccharide samples were detected and compared with that of Vc. Scavenging effects of samples against superoxide radical were positively correlated with their concentration in the range of 0.25–4.0 mg/mL (Figure 5C). The superoxide radical scavenging rates of PPP, PPP-1a, PPP-1b, PPP-2 and Vc at 0.25 mg/mL were high as 90.32%, 84.39%, 64.57%, 88.44% and 97.50%, respectively. At the high concentration of 4.0 mg/mL, the superoxide radical scavenging rates of PPP, PPP-1a, PPP-1b, PPP-2 and Vc reached to 94.55%, 91.15%, 92.47%, 99.66% and 98.98%, respectively, and the scavenging rates of PPP and PPP-2 showed no significant differences (*p* > 0.05) compared with that of Vc. The mechanism of superoxide radical scavenging activities may be related to the dissociation energy of O–H bond [59]. There was evidence that the presence of sulfate group in polysaccharides could enhance the superoxide radical scavenging activities due to the increase of electron-donating substituents in a saccharide ring, thus increasing electron density of the carbon atoms in the saccharide ring and facilitating liberation of hydrogen from O–H bond to stabilize superoxide anion [55,60]. In our present study, all the acidic polysaccharides showed strong superoxide radical scavenging activity, which is accordant with the results of the synthesized oversulphated derivatives of fucoidan extracted from *Laminaria japonica* [55]. The PPP and PPP-2 containing higher uronic acid also showed higher superoxide radical scavenging activity compared with PPP-1a and PPP-1b which contain no uronic acid, consisting with previous reports [61]. The effects of sulfate and carboxyl groups may be further confirmed by the desulfated and carboxyl-reduced derivatives of these polysaccharides in the future study, referring to our previous study [62].

#### 3.3.4. ABTS Radical Scavenging Activity

The assay of ABTS radical scavenging activity is commonly used in determining the total antioxidant activity of polysaccharides from natural products [34,63,64]. Herein, the results showed that ABTS radical scavenging activities of PPP, PPP-1a, PPP-1b and PPP-2 enhanced as their concentration increased (Figure 5D). Among the samples, the ABTS radical scavenging activity was determined to be in the order of PPP > PPP-2 > PPP-1a > PPP-1b. Particularly, at the concentration of 4.0 mg/mL, the scavenging rates of PPP, PPP-1a, PPP-1b, PPP-2 and Vc were 64.91%, 49.12%, 42.33%, 58.84% and 99.52%, respectively. And notably, PPP and PPP-2 had higher scavenging rates than PPP-1a and PPP-1b (*p* < 0.05). The results indicated that antioxidant activities of the polysaccharides determined by ABTS assay were positively correlated to those obtained by the DPPH radical assay, and that the scavenging ability obtained by ABTS assay was higher than that by DPPH radical assay. The ABTS assay may be more suitable than DPPH assay for evaluating the high water-soluble polysaccharides [15,65].

## 4. Conclusions

The extraction conditions for PPP were optimized by RSM with a BBD design in the present study. An extraction yield of 6.44 ± 0.06% was obtained by the optimal extraction conditions: ratio of water to raw material of 16.26 mL/g, extraction time of 2.89 h, extraction pH of 6.83, temperature of 50 °C and papain concentration of 0.15%. Three fractions of polysaccharides PPP-1a, PPP-1b and PPP-2 with the average molecular weights of 369.6, 41.73 and 57.76 kDa, respectively, were obtained from PPP by chromatography of FPA98Cl and Sepharose CL-6B columns. Further analysis showed that PPP-1a was composed of GalNAc, Gal and Fuc with Fuc as the main monosaccharide, PPP-1b contained Fuc as the only monosaccharide, and PPP-2 was composed of GlcA, GalNAc and Fuc with a nearly equal molar ratio. The sulfate contents of PPP, PPP-1a, PPP-1b and PPP-2 were 21.9%, 20.6%, 25.2% and 28.0%, respectively. The chemical analysis results along with the FT-IR spectra demonstrated that PPP, PPP-1a, PPP-1b and PPP-2 were all acidic polysaccharides. Furthermore, PPP, PPP-1a, PPP-1b and PPP-2 showed obvious scavenging activity of superoxide radical, DPPH radical, hydroxyl radical, and ABTS radical, which may be closely related to their structures such as monosaccharide compositions, and contents of carboxyl and sulfate groups. The results suggested that polysaccharides from sea cucumber *Phyllophorus proteus* may have protective effects as a potent antioxidant, which may provide valuable information for further research, enlarge the knowledge of sea cucumber polysaccharides.

## Figures and Tables

**Table 1:** Independent variables and their levels used for Box–Behnken design (BBD).

Factors	Code	Levels and Range		
		−1	0	1
A: ratio of extraction solvent to raw material (mL/g)	*X*_1_	10	15	20
B: extraction time (h)	*X*_2_	1.5	2.5	3.5
C: extraction pH	*X*_3_	5	6	7

**Table 2:** The Box–Behnken design matrix and the results of PPP extraction yield.

Experiment	A: Ratio of Extraction Solvent to Raw Material (mL/g)	B: Extraction Time (h)	C: Extraction pH	Yield (%)
1	−1	−1	0	3.48
2	1	−1	0	4.97
3	−1	1	0	5.12
4	1	1	0	5.86
5	−1	0	−1	2.71
6	1	0	−1	3.95
7	−1	0	1	5.12
8	1	0	1	5.97
9	0	−1	−1	2.78
10	0	1	−1	4.15
11	0	−1	1	5.18
12	0	1	1	6.24
13	0	0	0	6.83
14	0	0	0	6.03
15	0	0	0	6.32
16	0	0	0	6.56
17	0	0	0	6.14

**Table 3:** ANOVA for response surface quadratic model of PPP extraction.

Source	Sum of Squares	df	Mean Square	*F*-Value	*P*-Value
Model	26.71	9	2.97	49.15	<0.0001 **
*X*_1_	2.33	1	2.33	38.63	0.0004 **
*X*_2_	3.08	1	3.08	50.93	0.0002 **
*X*_3_	9.95	1	9.95	164.72	<0.0001 **
*X*_1_*X*_2_	0.14	1	0.14	2.33	0.1708
*X*_1_*X*_3_	0.038	1	0.038	0.63	0.4535
*X*_2_*X*_3_	0.024	1	0.024	0.40	0.5482
*X*_1_^2^	2.93	1	2.93	48.53	0.0002 **
*X*_2_^2^	1.97	1	1.97	32.65	0.0007 **
*X*_3_^2^	5.13	1	5.13	85.03	<0.0001 **
Residual	0.42	7	0.060		
Lack of fit	0.00415	3	0.001383	0.013	0.9976 ^a^
Pure error	0.42	4	0.10		
Cor Total	27.13	16			
*R*^2^ = 0.9844; Adj *R*^2^ = 0.9644; Pred *R*^2^ = 0.9735					

^a^ NS = non-significant. ** *p* < 0.01.

**Table 4:** Chemical compositions and physicochemical characteristics of PPP, PPP-1a, PPP-1b and PPP-2.

Item	PPP	PPP-1a	PPP-1b	PPP-2
Molecular weight (kDa)	/	369.6	41.73	57.76
Monosaccharide composition (mol)				
GlcA	1.00	ND ^a^	ND	1.00
GalNAc	2.34	1.00	ND	1.17
Gal	0.29	1.24	ND	ND
Fuc	9.41	8.04	1.00	1.77
Protein content (%)	3.62	0.14	0.01	0.02
Sulfate group content (%)	21.9	20.6	25.2	28.0
SO_3_^−^/COO^−^	/	/	/	3.32
Intrinsic viscosity (mL/g)	106.13	172.44	62.84	39.61
Specific rotation	−83.22°	−77°	−138.5°	−42.5°

^a^ ND: not determined.
